# A task-shared, collaborative care psychosocial intervention for improving depressive symptomatology among older adults in a socioeconomically deprived area of Brazil (PROACTIVE): a pragmatic, two-arm, parallel-group, cluster-randomised controlled trial

**DOI:** 10.1016/S2666-7568(22)00194-5

**Published:** 2022-10

**Authors:** Marcia Scazufca, Carina A Nakamura, Nadine Seward, Darío Moreno-Agostino, Pepijn van de Ven, William Hollingworth, Tim J Peters, Ricardo Araya

**Affiliations:** a Instituto de Psiquiatria, Hospital das Clinicas, Faculdade de Medicina, Universidade de Sao Paulo, Sao Paulo, Brazil; b Departamento de Psiquiatria, Faculdade de Medicina, Universidade de Sao Paulo, Sao Paulo, Brazil; c Health Service and Population Research, Institute of Psychiatry, Psychology and Neuroscience, King's College London, London, UK; d ESRC Centre for Society and Mental Health, King's College London, London, UK; e Centre for Longitudinal Studies, Social Research Institute, University College London, London, UK; f Health Research Institute, University of Limerick, Limerick, Ireland; g Health Economics Bristol, Population Health Sciences, Bristol Medical School, University of Bristol, Bristol, UK; h Bristol Dental School, University of Bristol, Bristol, UK

## Abstract

**Background:**

There is an urgent need to reduce the burden of depression among older adults in low-income and middle-income countries (LMICs). We aimed to evaluate the efficacy of a task-shared, collaborative care psychosocial intervention for improving recovery from depression in older adults in Brazil.

**Methods:**

PROACTIVE was a pragmatic, two-arm, parallel-group, cluster-randomised controlled trial conducted in Guarulhos, Brazil. Primary care clinics (clusters) were stratified by educational level and randomly allocated (1:1) to either enhanced usual care alone (control group) or to enhanced usual care plus the psychosocial intervention (intervention group), which involved a 17-week psychosocial programme based on psychoeducation and behavioural activation approaches. Individuals approached for the initial screening assessment were selected randomly from a list of individuals provided by the Health Secretariat of Guarulhos. Face-to-face baseline assessments were conducted among adults aged 60 years or older registered with one of the primary care clinics and identified with clinically significant depressive symptomatology (9-item Patient Health Questionnaire [PHQ-9] score ≥10). Community health workers delivered the programme through home sessions, supported by a dedicated tablet application. Masking of clinic staff and community health workers who delivered the intervention was not feasible; however, research assistants conducting recruitment and follow-up assessments were masked to trial allocation. The primary outcome was recovery from depression (PHQ-9 score <10) at 8-month follow-up. All primary analyses were performed by intention to treat with imputed data. Adaptations to the protocol were made due to the COVID-19 pandemic; recruitment and intervention home sessions were stopped, and follow-up assessments were conducted by telephone. This trial is registered with the ISRCTN registry, ISRCTN57805470.

**Findings:**

We identified 24 primary care clinics in Guarulhos that were willing to participate, of which 20 were randomly allocated to either the control group (ten [50%] clusters) or to the intervention group (ten [50%] clusters). The four remaining eligible clusters were kept as reserves. Between May 23, 2019, and Feb 21, 2020, 8146 individuals were assessed for eligibility, of whom 715 (8·8%) participants were recruited: 355 (49·7%) in the control group and 360 (50·3%) in the intervention group. 284 (80·0%) participants in the control group and 253 (70·3%) in the intervention group completed follow-up at 8 months. At 8-month follow-up, 158 (62·5%) participants in the intervention group showed recovery from depression (PHQ-9 score <10) compared with 125 (44·0%) in the control group (adjusted odds ratio 2·16 [95% CI 1·47–3·18]; p<0·0001). These findings were maintained in the complete case analysis. No adverse events related to the intervention were observed.

**Interpretation:**

Although the COVID-19 pandemic altered delivery of the intervention, the low-intensity psychosocial intervention delivered mainly by non-mental health professionals was highly efficacious in improving recovery from depression in older adults in Brazil. Our results support a low-resource intervention that could be useful to reduce the treatment gap for depression among older people in other LMICs.

**Funding:**

São Paulo Research Foundation and Joint Global Health Trials (UK Department for International Development, Medical Research Council, and the Wellcome Trust).

## Introduction

Depressive disorders impose a high burden on society and are considered to be the leading cause of disability globally.^1^ The 2019 National Health Survey in Brazil found a higher prevalence of depression among individuals aged 60 years or older (11·8%) than among younger adults (<60 years; 10·2%).^2^ In the National Health Survey 2013, approximately 80% of adults in Brazil identified with depression did not receive treatment.^3^ Among individuals who received treatment, access was markedly unequal, with those individuals aged 70 years or older with low educational attainment and living in the most disadvantaged areas of the country being particularly underserved.^3^ The gap in the provision of treatment for depression among older adults is especially concerning, given that the population is ageing fast, with around 15% (over 30 million) of Brazilians already aged 60 years and older.^4^

**Research in context**

**Evidence before this study**

We searched PubMed for randomised controlled trials on Dec 20, 2021, using the following search terms: ((((((“collaborative care”) OR (“task sharing”)) OR (“task shared”)) OR (“task shifting”)) OR (“task shifted”) or (psychosocial)) AND ((depression) OR (depressive))) AND (((older) OR (elderly)) OR (senior)). No language or date restrictions were applied. We found two studies in high-income countries that evaluated task-shared, collaborative care psychosocial interventions to treat older adults with depression in primary care (the IMPACT trial in the USA and the CASPER Plus trial in the UK). Results from these two trials showed an improvement in depressive symptomatology among participants at 4 months (CASPER Plus) and at 3, 6, and 12 months (IMPACT). We found only one randomised controlled trial investigating a collaborative care intervention for the treatment of depressive symptoms among older adults from a low-income and middle-income country, China; however, the intervention was based on antidepressant medication and did not include a psychosocial component. Our pilot study showed the feasibility and acceptability of a psychosocial intervention among users and providers in primary care clinics.

**Added value of this study**

To the best of our knowledge, we report the first large-scale cluster-randomised controlled trial (PROACTIVE) evaluating a task-shared, collaborative care psychosocial intervention for managing depressive symptomatology among older adults (aged ≥60 years) registered with primary care clinics in a low-income and middle-income country (Brazil). The intervention was efficacious at improving recovery from depression compared with enhanced usual care at both 8-month and 12-month follow-up assessments. The PROACTIVE trial innovatively used the most task-shared approach compared with previous collaborative care interventions for this population. The intervention was led by community health workers without a degree from higher education or formal training in mental health. An Android app was developed to support these workers during home sessions.

**Implications of all the available evidence**

Brief and low-intensive collaborative care psychosocial interventions delivered mainly by non-mental health professionals can be efficacious for older adults with depression in primary care across low-income and middle-income settings, as well as high-income settings. Future investigations should focus on how these interventions can be implemented at scale to help decrease the gap in the provision of treatment for depression in LMICs.

In 2021, Brazil had 4·3 psychiatrists and 32·5 clinical psychologists per 100 000 inhabitants, with unequal distribution across the country.^5^ If professionals working in the government-funded Brazilian Unified Health System, which caters for 75% of the older population, are considered alone, these rates decrease further to 2·7 psychiatrists and 17·9 clinical psychologists per 100 000 inhabitants.^6^ These figures are well below the necessary numbers to offer specialised mental health care to the entire population. To overcome the shortage of mental health specialists, WHO proposes integrating mental health care in primary care and transferring tasks (ie, task sharing) from mental health specialists to non-specialists or other non-medical service providers.^7^

Studies in high-income countries have shown the efficacy of treating older adults with depression in primary care with collaborative care models, including task sharing and stepped-care strategies.^8^, ^9^ The efficacy of this approach for the prevention of major depression among older adults with subsyndromal depressive symptoms was also found in a study from India.^10^ Low-intensity psychological therapies delivered at home and grounded on behavioural activation or problem-solving approaches featured prominently in these studies.

Collaborative care models for mental health problems involve a team following an agreed and monitored care plan.^11^ Task-sharing strategies involve transferring clinical duties to trained and supervised non-medical health workers, such as community health workers (CHWs). Stepped-care models involve deploying resources according to the needs of patients—ie, individuals with severe conditions receive more intense interventions than do those with mild problems.

Given social and cultural disparities, as well as differences in health-care systems, it is not appropriate to assume that models developed and tested in high-income countries can be successfully transferred to Brazil or other low-income and middle-income countries (LMICs). Programmes for adults (aged ≥16 years) with depression have applied some of the ideas contained in these models and shown good results in LMICs; however, to date, no studies have been conducted in older adults (aged ≥60 years).^12^, ^13^, ^14^ Our pilot of a psychosocial collaborative, stepped-care, and task-shared intervention,^15^ supported by technology^16^ targeting older adults with depression in low-income neighbourhoods in São Paulo, Brazil, proved feasible and acceptable to users and providers in primary care clinics.

The Family Health Strategy^17^ in Brazil offers suitable conditions to implement task-shared, collaborative care programmes to treat older adults with depression. In this primary care model, staff work collaboratively in primary health-care clinics, known as Unidades Básicas de Saúde, which are divided into family health teams (FHTs). Each FHT comprises a minimum of a family physician, a nurse, a nurse assistant, and around six CHWs, who are responsible for providing comprehensive care to up to 3500 inhabitants of the catchment area.^17^ CHWs are trained non-health professionals with a central role in this model, visiting households regularly to identify health problems and to monitor ongoing treatments. In 2020, over 43 000 FHTs were active in Brazil, covering 64% of the population mainly in socioeconomically deprived areas of the country.^18^ However, mental health care is still not adequately integrated into the Family Health Strategy.

This trial builds on our successful pilot study.^15^, ^19^ We aimed to evaluate the efficacy of a community psychosocial intervention, managed mostly by CHWs supported by technology,^16^ for improving recovery from depression among older adults in a socioeconomically deprived area of Brazil. We also reviewed the implications of the COVID-19 pandemic on this trial and evaluated how deviations from the research protocol were addressed.

## Methods

### Study design and participants

PROACTIVE was a pragmatic, two-arm, parallel-group, cluster-randomised controlled trial conducted in Guarulhos, Brazil. Guarulhos is the second largest city in the state of São Paulo, and part of the São Paulo conurbation. The population of Guarulhos, which is estimated to be 1·4 million people,^20^ live in diverse socioeconomic conditions and around 31% are served by 39 primary care clinics with FHTs.^18^

Eligible trial clusters were primary care clinics in Guarulhos that were willing to participate and that adhered to the Brazilian Family Health Strategy with at least four FHTs. To reflect what would be the most likely approach if rolled out in practice, the intervention was delivered in the relevant cluster by CHWs selected by each site manager.

Eligible participants were adults aged 60 years or older registered with one of the participating FHTs and identified with clinically significant depressive symptomatology (9-item Patient Health Questionnaire [PHQ-9] score ≥10).^21^ Individuals who had cognitive, communication, visual, or hearing problems; were unable to engage in the trial for 12 months; were identified with an acute risk of suicide at screening assessment; or had another person in their household included in the study, were excluded from the trial. Written informed consent was obtained from individuals before starting screening and baseline assessments. If the screening interview was conducted by telephone, verbal consent was recorded. This study was approved by the Ethics Committee of Universidade de Sao Paulo Medical School (CEP FMUSP number 2.836.569) and authorised by the Guarulhos Health Secretary. The trial protocol has been published previously.^22^

### Randomisation and masking

A statistician (TJP) not involved in the recruitment process was responsible for the randomisation process (ie, the allocation of primary care clinics and selection of FHTs). Eligible primary care clinics were stratified by educational level and randomly allocated (1:1) to either enhanced usual care alone (control group) or to enhanced usual care plus the psychosocial intervention (intervention group). Primary care clinics were randomly allocated by use of computer-generated random numbers to each trial group within each of two equal-sized strata, defined by whether or not the percentage of individuals in each cluster who had either no formal education or had only completed a literacy programme for adults was above or below the median. Eligible clusters not assigned were kept as reserves.

Masking of clinic staff and CHWs who delivered the intervention was not feasible. Trial allocation was only revealed to primary care clinic managers just before recruitment of participants commenced. Research assistants conducting recruitment and follow-up assessments were masked to trial allocation. Whenever possible, research assistants did not interview the same participant more than once.

### Procedures

Recruitment was planned to be conducted by independent research assistants in two waves, aiming to enrol 720 participants in each wave. Individuals approached for the initial screening assessment were selected randomly from a list of individuals provided by the Health Secretariat of Guarulhos with the names of all eligible adults aged 60 years or older who were registered with the participating primary care clinics. The PHQ-9 screening assessment was mainly conducted at home, but telephone calls were also used when individuals were not found to be at home after several visits. Following this screening assessment, individuals identified with clinically significant depressive symptomatology (PHQ-9 score ≥10) were invited to the face-to-face baseline assessment, which also took place at home. Data were collected using our own software—the PROACTIVE app (version 2.0)—installed on tablet computers. The follow-up assessments at 8 months and 12 months after the baseline assessment were planned to be conducted within a 4-week window period via home visits.

Both groups received enhanced usual care. Managers at primary care clinics were informed about all participants included in the study. Managers could share information about the depressive symptomatology collected by the research assistants with members of the FHT and discuss a treatment plan as part of enhanced usual care. A tight safety protocol for suicidal risk was also part of the enhanced usual care. Beyond this intervention, the research did not affect the usual care offered by the FHT, including clinical decisions related to depression (eg, prescription of antidepressants, consultation with family doctors or mental health specialists, and routine home visits by CHWs to all households registered with the FHT).

The psychosocial intervention consisted of a 17-week programme delivered by CHWs during a series of scheduled home sessions (appendix p 1). We chose home sessions to improve participant adherence to the intervention and to assist older adults who had difficulties travelling or were confined to bed. The intervention content was based on psychoeducation^23^ and behavioural activation.^24^ Psychoeducation involved education about symptoms of depression, relapse prevention strategies, and simple ways to cope with depressive symptoms and associated problems, while behavioural activation involved education about the importance of engaging in pleasant or meaningful activities and positive interactions with the environment. Sessions were exclusively delivered with the support of the PROACTIVE app installed on the Android tablets of CHWs.^16^ CHWs used the app to present all intervention content to participants. A total of 24 short videos presented by a narrator and illustrated by animations were developed for this intervention, with contents related to the session. The app recorded all participant responses, such as PHQ-9 responses, mood ratings, type of and adherence to homework, and chosen strategies (eg, type of pleasant and meaningful activities to work on). This data collection facilitated the review and discussion between CHWs and clinical supervisors. The app also included rule-based support to clinical decision making by the CHW and empowered CHWs to conduct the home sessions with participants independently.

The intervention was divided into an initial phase of 3 weeks, followed by a second phase of 14 weeks. The first phase involved three weekly home sessions focused on psychoeducation to all participants. PHQ-9 was assessed at each of these three sessions. Once the initial phase had been completed, participants were assigned to second-phase regimens of either low or high intensity, focusing on behavioural activation and techniques for relapse prevention. The level of intensity was decided according to the level of depressive symptomatology measured by the PHQ-9 score at the second and third sessions. If an improvement from baseline was observed in both the second and third sessions (ie, PHQ-9 score <10), participants received the low-intensity regimen, ie five additional sessions (three sessions every 2 weeks followed by two sessions per month). If no improvement was observed in either or both sessions two and three (PHQ-9 score ≥10), participants were assigned to the high-intensity regimen and received eight additional sessions (six sessions per week, followed by two sessions per month).

120 CHWs (three per FHT) received 3 days of training on how to deliver the intervention, and group supervision sessions with a mental health specialist (clinical supervisor) were held on a weekly basis to discuss cases and to receive technical and clinical support. Each CHW delivered home sessions to approximately three participants. Any further care needed by a participant at the primary care clinics or with mental health specialists was decided among the FHT members as part of usual care. A documentary showing the settings and how the intervention was conducted by community health workers is available online.

Assessments at the 8-month and 12-month follow-up visits included depressive symptomatology as a continuous measure (PHQ-9 total scores^22^), anxiety measured by the seven-item Generalized Anxiety Disorder (GAD-7) assessment scores,^25^ health-related quality of life measured by the European Quality of Life Five-level version (EQ-5D-5L),^26^ and capability wellbeing measured by the ICEpop CAPability measure for Older people (ICECAP-O).^27^ PHQ-9 is a measure of depressive symptomatology,^21^ which is able to detect changes over time.^28^ This measure is widely used and validated among the Brazilian population and our study population.^29^ GAD-7 is commonly used to measure symptomatology of general anxiety.^25^ EQ-5D-5L is a generic, preference-based outcome that measures health-related quality of life,^26^ and ICECAP-O measures capability wellbeing in older adults.^27^ Evidence of validity in differentiating changes in depressive symptomatology was shown for both EQ-5D-5L and ICECAP-O measures.^30^ Given that no value set for the Brazilian population has been developed for these measures to date, we used the EQ-5D-5L values for the Uruguayuan population^31^ and ICECAP-O tariffs for the UK population.^32^

Data on adverse events were collected using a specially designed form. If events were not spontaneously reported during the study, they were asked about during the home visits at weeks 3, 7, 13, and 17, at the last telephone session, and at both follow-up assessments. If an event was reported, then the intervention coordinator ensured completion of the forms regarding the intensity of the event and whether it was related to the intervention, before the principal investigator (RA, not involved with clinical supervision and data collection) judged if the serious adverse event was related to the trial intervention.

Due to the onset of the COVID-19 pandemic in Brazil in March, 2020, recruitment and all intervention activities that included face-to-face contact with participants were stopped from March 12, 2020. This situation resulted in four main deviations from the initial trial protocol reported following the CONSERVE guidelines.^33^ First, recruitment of new participants ceased before the planned second wave of recruitment. Second, participants who had not completed all programme sessions and had not formally withdrawn from the study were offered two telephone sessions instead of the remaining programme sessions. The clinical supervisors of the CHWs were trained to deliver the telephone sessions. These structured sessions lasted around 30 min, with contents based on psychoeducation and behavioural activation approaches. All participants were offered two telephone sessions independently of the number of face-to-face sessions that they had received before. Third, the 8-month follow-up started on Jan 17, 2020, and contacted participants were assessed face to face until March 12, 2020. Thereafter, all 8-month and 12-month follow-up assessments were carried out by telephone. Given that remote interviews were not initially planned, we contacted managers and CHWs at the primary care clinics to confirm and update participants’ telephone numbers; we delivered a letter to participants to remind them about the two follow-up assessments; if they were at home, we confirmed and updated telephone numbers; and we offered a short version of the follow-up assessment only for individuals who declined to participate in a long interview by telephone. The administration of the Behavioural Activation for Depression Scale-Short Form^34^ required visual aids for its completion by the study population. For this reason, data collection for this outcome was discontinued.

### Outcomes

All outcomes pertained to the individual level. The primary outcome was the proportion of participants who had recovered from depression, defined as a PHQ-9 score of less than 10, at the 8-month follow-up assessment. Secondary outcomes included the proportion of participants with depression recovery (PHQ-9 total scores <10) at 12-month follow-up, plus the following measures at 8-month and 12-month follow-up: depressive symptomatology as a continuous measure (PHQ-9 total scores^21^), anxiety (GAD-7 assessment scores),^25^ health-related quality of life (EQ-5D-5L scores),^26^ and capability wellbeing (ICECAP-O scores).^27^

### Statistical analysis

To detect a 15 percentage point difference (25% *vs* 40%)^8^, ^15^ in depression recovery rates between the control group and the intervention group at 8-month follow-up, with 85% power and two-sided 5% significance, 374 individuals were required. With 15% attrition, this number was increased to 440 participants. Assuming an intracluster correlation coefficient of 0·03 based on the pilot study, and 72 participants available in each cluster, the corresponding design effect was 3·13, resulting in an inflated total study population of 1378 individuals (3·13 × 440), which for a cluster size of 72 required 19·13 clusters. Therefore, we aimed to recruit a total of 1440 participants from 20 clusters, comprising 72 participants per cluster. Consequently, we kept four clusters (two per stratum) in reserve in case any of the randomly selected 20 clusters subsequently declined to participate or had to withdraw. Recruitment was stopped on March 12, 2020, due to the COVID-19 pandemic; therefore, only data gathered from the first wave of recruitment, representing half of the total sample size planned, are reported in this analysis. Descriptive statistics based on complete cases only were used to explore any imbalances between treatment groups at baseline. All primary analyses were performed by intention to treat with imputed data. Analyses based on complete cases only were also carried out to compare findings. At both the 8-month and 12-month follow-up visits, the analyses for primary and secondary outcomes adhered to the statistical analysis plan and were conducted according to randomisation group.

All regression analyses used random-effects models with a random intercept to account for clustering, and were adjusted for stratification (above or below the median proportion of adults aged ≥60 years with no formal education) and the baseline score of the corresponding outcome. We assessed the primary outcome of recovery from depression at 8-month follow-up and the secondary outcome of recovery from depression at 12-month follow-up using logistic regression models that assumed linearity in the logit of the outcome for all explanatory variables. Linear regression models, which assumed that residuals of the model were normally distributed, were used to evaluate all other secondary outcomes measured at both follow-up assessments.

We investigated prespecified subgroup analyses using likelihood ratio tests for interactions assessed at both follow-up assessments, using product terms between the intervention group and the following variables: gender, age, educational level groups based on the Brazilian schooling system, comorbid physical illness (ie, diabetes, hypertension, or both), and baseline PHQ-9 score. We also ran a model for the primary outcome, adjusting for elapsed time between consenting into the trial and the first follow-up assessment at 8 months.

We used the complier average causal effect (CACE) analysis, using an instrumental variable estimator and accounting for clustering within the different clusters, to estimate the effect of the number of sessions attended on depressive symptoms.^35^ The CACE parameter measures the effect of the intervention among the subgroup of participants who complied with the assigned treatment. To this effect, we primarily used a four-session threshold to conduct a CACE analysis with the PHQ-9 score at 8 months and 12 months as the outcome. We also conducted sensitivity analyses using three-session and five-session thresholds.

Missing data were replaced using multiple imputation by chained equations, as implemented in the MI command in Stata (version 15) under the assumption that data were missing at random. Data were imputed for the analyses of primary and secondary outcomes, as well as for the CACE analyses. Details of the methods used for the missing data analyses, including sensitivity analyses and results comparing estimates using complete cases versus imputed data, are provided in the appendix (p 2).

Regression diagnostics were run for both the logistic regression and the linear regression models. The Box-Tidwell test was run after the logistic regression models to test whether the logit transform was a linear function of the predictors for the different models. Normality assumptions for all linear regression models were evaluated by examining the residual plots after running the regression commands, using the post-estimation command predict r, resid followed by the pnorm and qnorm commands in Stata.

Additional sensitivity analyses due to issues surrounding the COVID-19 pandemic were conducted. To consider any potential influences that the type of follow-up interview (ie, telephone *vs* face to face) might have had, we ran a mixed-effects linear regression model comparing continuous outcomes measures (ie, PHQ-9, GAD-7, EQ-5D-5L, and ICECAP-O) between the control group and intervention group at the 8-month follow-up assessment, adjusting for interview type. To investigate differences in outcomes by type of interview, we ran ANOVA models for each of the different outcomes by treatment type (adjusting for baseline scores of associated outcome measure). These analyses were not needed for the 12-month follow-up because all such interviews were conducted by telephone.

Because it was possible that sessions delivered by telephone were also effective in reducing symptoms of depression when face-to-face sessions were no longer possible due to the COVID-19 pandemic, we performed CACE analyses at 8 months and 12 months. The threshold of four sessions included participants who had received four sessions, regardless of whether these were by telephone or in person.

To ensure that our findings were robust to other approaches to analyse binary outcomes with a cluster-randomised trial, we ran two additional post-hoc analyses using complete data only. First, we used a modified Poisson regression model, as suggested by Zou and Donner.^36^ Second, we ran a mixed-effects logistic regression using the outcome of whether a participant had a 50% reduction in PHQ-9 scores between baseline and each follow-up visit.

All analyses were done with Stata (version 15.0). This trial is registered with the ISRCTN registry, ISRCTN57805470.

### Role of the funding source

The funders of the study had no role in study design, data collection, data analysis, data interpretation, or writing of the report.

## Results

**Figure 1:**
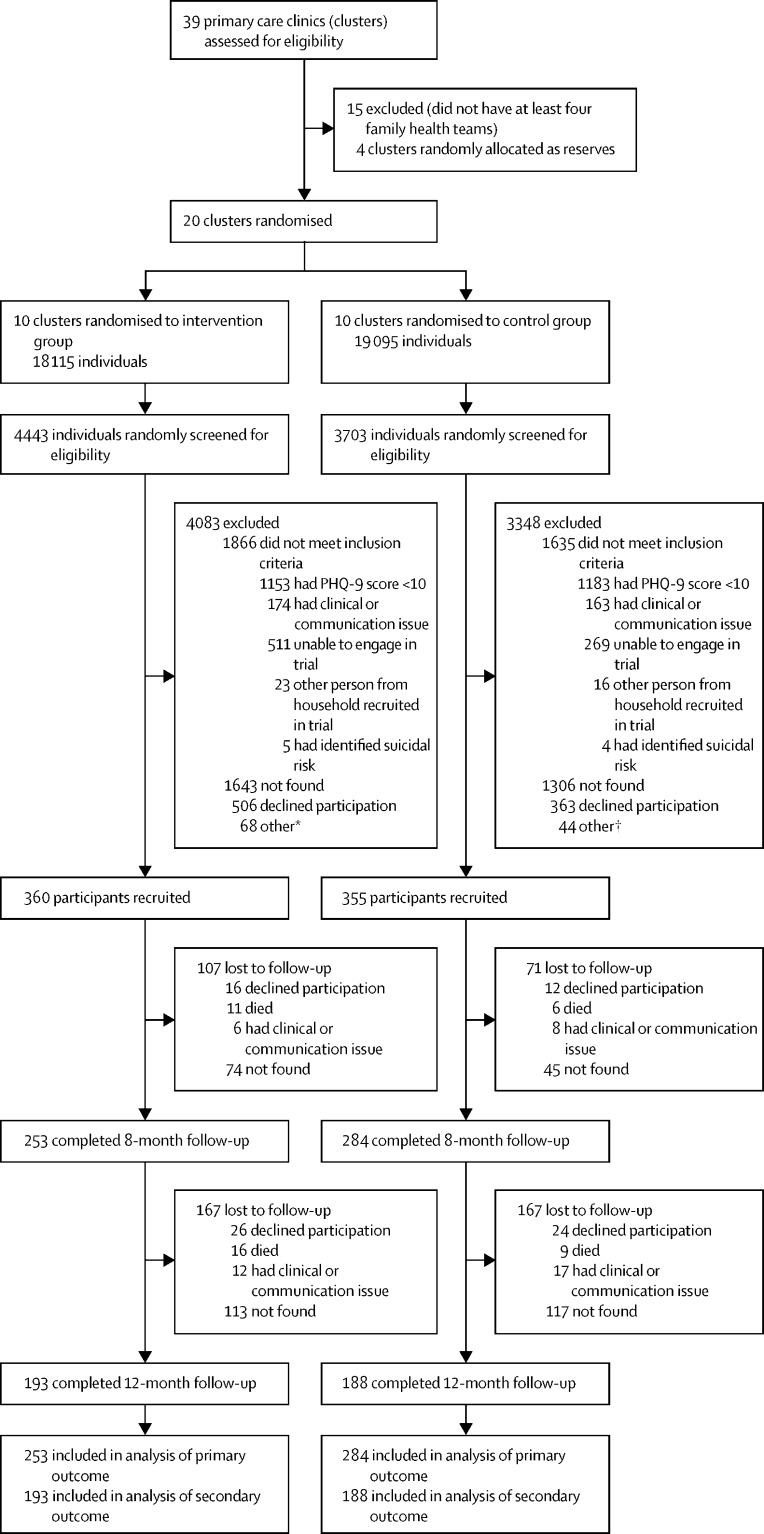
Trial profile PHQ-9=9-item Patient Health Questionnaire. *Community health workers reached quota (recruitment by family health team complete [n=68]). †Community health workers reached quota (n=38) and assessment not approved by quality control (n=6).

We identified 24 primary care clinics in Guarulhos that were willing to participate and that adhered to the Brazilian Family Health Strategy with at least four FHTs. 24 eligible primary care clinics were stratified by educational level and 20 were randomly allocated (1:1) to either enhanced usual care alone (control group; ten [50%] clusters) or to enhanced usual care plus the psychosocial intervention (intervention group; ten [50%] clusters). Five primary care clinics were randomly allocated to each trial group within each of two equal-sized strata, defined by whether or not the percentage of individuals in each cluster who had either no formal education or had only completed a literacy programme for adults was above or below the median. Of the 20 allocated primary care clinics, 11 had four FHTs, eight had five, and one had six; for the clinics with five or six FHTs, four FHTs were selected at random within each primary care clinic separately. The first wave of recruitment took place between May 23, 2019, and Feb 21, 2020. Recruitment was planned to be conducted by independent research assistants in two waves, aiming to enrol 720 participants in each wave. Individuals approached for the initial screening assessment were selected randomly from a list of individuals provided by the Health Secretariat of Guarulhos with the names of all 37 210 older adults registered with the 20 primary care clinics participating in the study. Of the 8146 individuals screened for eligibility, 3501 (43·0%) did not meet eligibility criteria (2336 did not have depressive symptomatology), 869 (10·7%) declined participation, and 2949 (36·2%) could not be contacted (figure 1). A total of 715 (8·8%) participants were recruited, 355 (49·7%) in the control group and 360 (50·3%) in the intervention group (figure 1). 36 participants were recruited in each primary care clinic, with the exception of two primary care clinics allocated to the control group, in which 33 and 34 participants were included.

Due to the COVID-19 pandemic, recruitment was halted on March 12, 2020, after the first phase of recruitment. Consequently, 143 participants (39·7%) in the intervention group who had not completed all programme sessions and had not formally withdrawn from the study were offered two telephone sessions instead of the remaining face-to-face programme sessions. 102 (81%) of 126 contacted participants were assessed face-to-face for their 8-month assessment until March 12, 2020. Thereafter, all 8-month and 12-month follow-up assessments were carried out by telephone.

A total of 537 (75·1%) recruited participants were followed up at 8 months (figure 1). A greater proportion of participants was lost to follow-up in the intervention group (107 participants [29·7%]) than in the control group (71 [20·0%]). At 12-month follow-up, 381 (53·3%) participants were followed up, with similar proportions lost to follow-up at 12 months in the intervention group (167 participants [46·4%]) and in the control group (167 [47·0%]; figure 1). Baseline characteristics remained balanced at both follow-up visits (appendix pp 9–10).

Descriptive statistics of baseline characteristics suggest that there were no major differences between the control group and the intervention group (table 1). The majority of participants were women aged 60–69 years from a low socioeconomic background; most participants had up to 4 years of education and a monthly personal income of up to the minimum wage. Prevalence of comorbidity (ie, hypertension or diabetes) was high (table 1).

**Table 1:** Baseline characteristics in the intervention group and the control group

		**Intervention group (n=360)**	**Control group (n=355)**
Sex			
	Female	268 (74·4%)	262 (73·8%)
	Male	92 (25·6%)	93 (26·2%)
Age group, years			
	60–69	223 (61·9%)	217 (61·1%)
	70–79	110 (30·6%)	104 (29·3%)
	≥80	27 (7·5%)	34 (9·6%)
Time spent in education, years			
	0	72 (20·1%)	65 (18·4%)
	1–4	173 (48·2%)	180 (50·8%)
	5–8	70 (19·5%)	71 (20·1%)
	>8	44 (12·3%)	38 (10·7%)
	Data missing	1 (0·3%)	1 (0·3%)
Monthly personal income, minimum wage*			
	≤1	263 (76·0%)	255 (75·7%)
	>1 to 2	61 (17·6%)	55 (16·3%)
	>2	22 (6·4%)	27 (8·0%)
	Data missing	14 (3·9%)	18 (5·1%)
Hypertension (self-reported)		274 (76·1%)	268 (75·5%)
Diabetes (self-reported)		148 (41·1%)	146 (41·1%)
Receiving pharmacological treatment for depression (self-reported)			
	No	297 (83·2%)	298 (84·4%)
	Yes	60 (16·8%)	55 (15·6%)
	Data missing	3 (0·8%)	2 (0·6%)
Bereavement (death of a family member or close friend in the previous 12 months)			
	No	148 (41·1%)	162 (45·8%)
	Yes	212 (58·9%)	192 (54·2%)
	Data missing	0	1 (0·3%)
PHQ-9 score†		16·04 (4·58)	16·34 (4·69)
GAD-7 score‡		10·16 (6·06)	9·43 (6·16)
	Data missing	1 (0·3%)	3 (0·8%)
EQ-5D-5L score§		0·759 (0·206)	0·753 (0·197)
	Data missing	2 (0·6%)	1 (0·3%)
ICECAP-O score¶		0·628 (0·197)	0·639 (0·198)
	Data missing	4 (1·1%)	1 (0·3%)

Data are n (%) or mean (SD). PHQ-9=9-item Patient Health Questionnaire. GAD-7=seven-item Generalized Anxiety Disorder assessment. EQ-5D-5L=European Quality of Life Five-level version. ICECAP-O=ICEpop CAPability measure for Older people.

* In 2019, the minimum wage in Brazil was 998 Brazilian real (approximately US$253).

† PHQ-9 scores range from 0 to 27, with higher scores representing more severe symptoms of depression.

‡ GAD-7 scores range from 0 to 21 with higher scores representing more severe symptoms of anxiety.

§ EQ-5D-5L scores range from −0·264 to 1·000, with higher scores representing higher quality of life.

¶ ICECAP-O scores range from 0 to 1, with higher scores representing greater levels of capability wellbeing.

**Figure 2:**
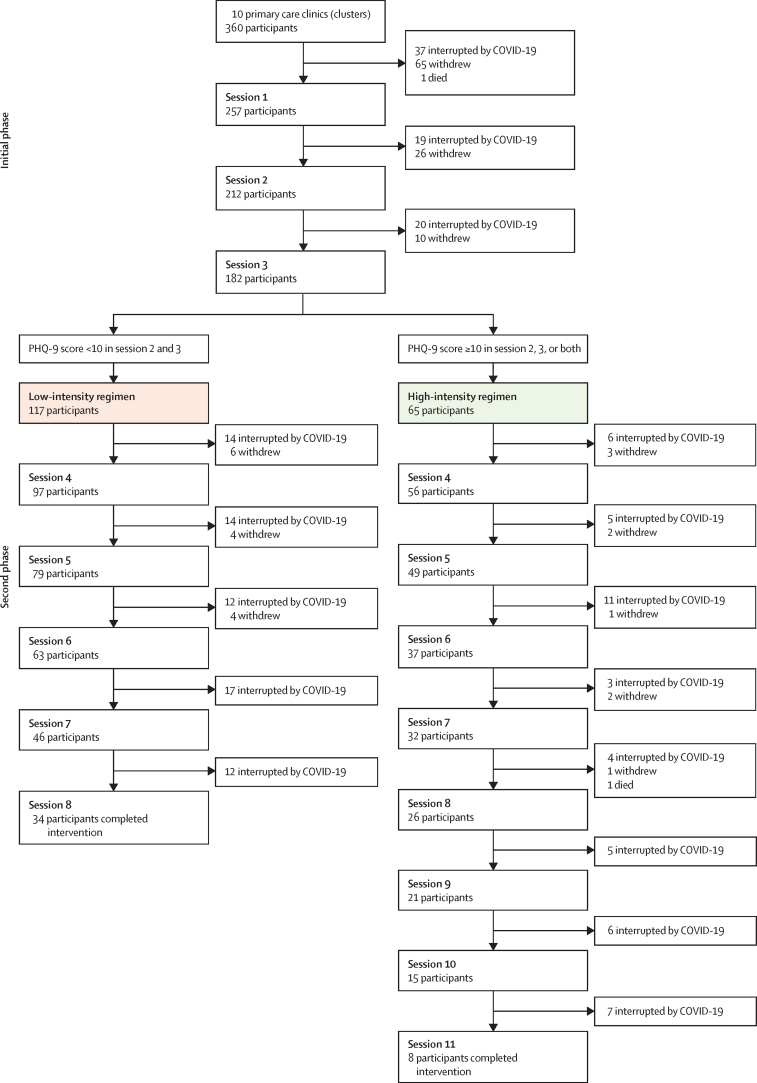
Flow of participants in the intervention group PHQ-9=9-item Patient Health Questionnaire.

Among the 360 participants in the intervention group, 182 (50·6%) completed the initial phase (first three sessions), 101 (28·1%) withdrew from the intervention, the programme was interrupted because of the COVID-19 pandemic for 76 (21·1%), and one (0·3%) died before the first session (figure 2). Among individuals who completed the initial phase, 117 (64·3%) were assigned to the low-intensity regimen and 65 (35·7%) to the high-intensity regimen. 34 (29·1%) participants in the low-intensity regimen and eight (7·7%) in the high-intensity regimen received all home sessions (figure 2). The onset of the COVID-19 pandemic was the main reason for participants not completing the second phase of the intervention (116 [63·7%] participants), followed by withdrawal from the intervention (23 [12·6%]). One (0·5%) participant in the high-intensity group died.

Results from the primary analysis showed a significant effect of the psychosocial intervention on recovery from depression (PHQ-9 score <10) at the 8-month follow-up assessment, with 158 (62·5%) participants in the intervention group recovering from depression compared with 125 (44·0%) in the control group (adjusted odds ratio 2·16 [95% CI 1·47–3·18]; p<0·0001; table 2). These findings were maintained in the complete case analysis (appendix p 6). A significant effect of the intervention on recovery from depression was also observed at the 12-month follow-up assessment (2·33 [1·45–3·71]; p<0·0001; table 2).

**Table 2:** Primary and secondary outcomes at 8-month and 12-month follow-up

	**Intervention group**	**Control group**	OR (95% CI)*†	**p value**
Primary outcome: recovery from depression at 8 months‡	158/253 (62·5%)	125/284 (44·0%)	2·16 (1·47–3·18)	<0·0001
Secondary outcome: recovery from depression at 12 months	115/193 (59·6%)	77/188 (41·0%)	2·33 (1·45–3·71)	<0·0001

Data are n/N (%) unless otherwise indicated. OR=odds ratio. PHQ-9=9-item Patient Health Questionnaire.

* ORs and 95% CIs were calculated using random-effects logistic regression models.

† All estimates had missing data imputed by intervention group using multiple imputation by chained equations models that included baseline PHQ-9 scores, stratification (median proportion of adults aged ≥60 years with no formal education), and predictors of missingness (appendix p 2).

‡ Defined as a PHQ-9 score <10.

PHQ-9 scores were lower in the intervention group than in the control group at both 8 months and 12 months (table 3). Anxiety symptomatology levels (GAD-7 scores) were also lower in the intervention group than in the control group at both follow-up assessments. Health-related quality of life (EQ-5D-5L scores) were significantly better in the intervention group compared to the control group at 8 months, but not at 12 months. No significant differences in capability wellbeing (ICECAP-O scores) were found between groups at either follow-up assessment. Estimates for the primary and secondary outcomes were similar for complete case analyses and analyses with imputed data (appendix pp 6–7).

**Table 3:** Additional secondary outcomes at 8-month and 12-month follow-up

		**8-month follow-up**				**12-month follow-up**			
		Intervention group (n=253)	Control group (n=284)	Adjusted difference in means (95% CI)*†	p value	Intervention group (n=193)	Control group (n=188)	Adjusted difference in means (95% CI)*†	p value
PHQ-9 score‡		253 (100·0%)	284 (100·0%)	..	..	193 (100·0%)	188 (100·0%)	..	..
	Mean (SD)	8·82 (7·23)	11·52 (6·92)	−2·52 (−3·69 to −1·36)	<0·0001	9·32 (7·28)	11·56 (6·92)	−2·32 (−4·08 to −0·55)	0·011
	Median (IQR)	7 (3–13)	11 (6–17)	..	..	8 (4–15)	12 (6–16)	..	..
GAD-7 score§		231 (91·3%)	247 (87·0%)	..	..	170 (88·1%)	168 (89·4%)	..	..
	Mean (SD)	7·88 (6·44)	8·70 (5·97)	−1·21 (−2·29 to −0·12)	0·030	7·62 (6·04)	9·09 (6·45)	−2·09 (−3·30 to −0·89)	0·0010
	Median (IQR)	7 (2–14)	8 (4–14)	..	..	7 (2–12)	9 (3–15)	..	..
EQ-5D-5L score¶		242 (95·7%)	265 (93·3%)	..	..	175 (90·7%)	175 (93·1%)	..	..
	Mean (SD)	0·831 (0·164)	0·806 (0·186)	0·030 (0·003 to 0·056)	0·029	0·818 (0·200)	0·809 (0·170)	0·003 (−0·028 to 0·035)	0·83
	Median (IQR)	0·876 (0·750–0·945)	0·870 (0·745–0·929)	..	..	0·883 (0·750–0·961)	0·852 (0·723–0·927)	..	..
ICECAP-O score‖		239 (94·5%)	261 (91·9%)	..	..	175 (90·7%)	176 (93·6%)	..	..
	Mean (SD)	0·661 (0·198)	0·642 (0·187)	0·014 (−0·015 to 0·043)	0·34	0·687 (0·213)	0·641 (0·174)	0·039 (−0·011 to 0·090)	0·12
	Median (IQR)	0·695 (0·556–0·793)	0·658 (0·556–0·758)	..	..	0·722 (0·562–0·853)	0·670 (0·556–0·761)	..	..

Data are n (%), unless otherwise indicated. PHQ-9=9-item Patient Health Questionnaire. GAD-7=seven-item Generalized Anxiety Disorder assessment. EQ-5D-5L=European Quality of Life Five-level version. ICECAP-O=ICEpop CAPability measure for Older people.

* Difference in means were estimated using linear regression models with random effects, adjusted for the baseline assessment of the corresponding outcome and the stratified variable of education (median proportion of adults aged ≥60 years with no formal education).

† All estimates had missing data imputed separately, by intervention group, using multiple imputation by chained equations models that included predictors of missingness (appendix p 2), stratification (median proportion of adults aged ≥60 years with no formal education), baseline PHQ-9 scores, and any imbalances in outcome measure at baseline (GAD-7 only).

‡ PHQ-9 scores range from 0 to 27, with higher scores representing more severe symptoms of depression.

§ GAD-7 scores range from 0 to 21 with higher scores representing more severe symptoms of anxiety.

¶ EQ-5D-5L scores range from −0·264 to 1·000, with higher scores representing higher quality of life.

‖ ICECAP-O scores range from 0 to 1, with higher scores representing greater levels of capability wellbeing.

Estimates from the CACE analysis, which evaluated the effect of completing at least four face-to-face sessions (the prespecified minimum therapeutic dose) after imputing missing data, showed an improvement in mean PHQ-9 scores in participants who completed four sessions compared with those who completed fewer than four sessions, at both 8 months and 12 months (table 4). At both follow-up assessments, this difference in mean scores was smaller when the threshold for compliance was reduced to three sessions, and was larger when it was raised to five sessions. Adjusting for the number of days elapsed from baseline to the first follow-up at 8 months had no appreciable effect on the relevant analyses, and there was no evidence of differential effects according to the prespecified subgroup analyses using likelihood ratio tests (appendix pp 10–11).

**Table 4:** Complier average causal effects analysis of mean PHQ-9 scores at 8-month and 12-month follow-up

	**8-month follow-up**		**12-month follow-up**	
	Adjusted difference in mean PHQ-9 scores (95% CI)	p value	Adjusted difference in mean PHQ-9 scores (95% CI)	p value
Three sessions	−5·09 (−7·40 to −2·77)	<0·0001	−4·93 (−7·89 to −1·97)	0·0010
Four sessions	−6·05 (−8·85 to −3·26)	<0·0001	−5·86 (−9·41 to −2·31)	0·0020
Five sessions	−7·23 (−10·61 to −3·85)	<0·0001	−7·00 (−11·27 to −2·74)	0·0020

PHQ-9 scores range from 0 to 27, with higher scores representing more severe symptoms of depression. All models are adjusted for baseline PHQ-9 scores and stratification. 95% CIs were adjusted for clustering. All estimates had missing data imputed separately, by intervention group, using multiple imputation by chained equations models that included predictors of missingness (appendix p 2), stratification (median proportion of adults aged ≥60 years with no formal education), and baseline PHQ-9 scores. PHQ-9=9-item Patient Health Questionnaire.

Due to the onset of the COVID-19 pandemic, most of the follow-up interviews at 8 months had to be conducted by telephone (435 [81·0%] of 537 participants), instead of in person (102 [19·0%]) as originally intended. At the 8-month follow-up assessment, a slightly larger proportion of participants in the intervention group were contacted by telephone than in the control group (214 [84·6%] of 253 participants *vs* 221 [77·8%] of 284 participants). However, there were no differences in outcomes between participants in both groups whose 8-month assessments were conducted by telephone versus in person, except for health-related quality of life (EQ-5D-5L scores); those followed up by telephone reported lower EQ-5D-5L scores than did those contacted in person (appendix p 11). No differences were found in adjusted models including or not including type of follow-up visit (appendix p 11).

At the onset of the COVID-19 pandemic, face-to-face sessions were stopped, and up to two extra sessions were delivered by telephone. To test whether delivering the sessions by telephone influenced PHQ-9 scores, additional CACE analyses were conducted at both 8 months and 12 months. These analyses included participants who had completed four or more sessions delivered in person or by telephone. Results from the additional CACE analyses indicated similar improvements in mean PHQ-9 scores between participants who had completed four sessions and those who had completed fewer than four sessions (appendix p 7). Our findings were robust in the modified Poisson approach and with use of the outcome of a 50% reduction in PHQ-9 scores between baseline and each of the follow-up visits (appendix p 12).

Among the 360 participants in the intervention group, three (0·8%) individuals reported suicidal ideation (without suicide attempt) while receiving home sessions, and four (1·1%) while receiving sessions by telephone. Data from follow-up assessments showed that hospitalisations were reported by 33 (9·2%) participants in the intervention group and by 36 (10·1%) in the control group during the study. 16 (4·4%) deaths in the intervention group and nine (2·5%) deaths in the control group were also reported, all due to natural causes. None of these events were related to the trial interventions according to the protocol assessing adverse events.

## Discussion

To our knowledge, the PROACTIVE trial is the first large-scale cluster-randomised controlled trial of a task-shared, collaborative care psychosocial intervention for older adults with depressive symptomatology in primary care clinics in Latin America. The intervention showed a substantial increase in the proportion of participants who recovered from depression in the intervention group compared with those in the control group at both 8-month and 12-month follow-up visits. Participants in the intervention group also showed lower levels of anxiety symptomatology than did those in the control group, at both 8 months and 12 months. Although there was a trend in the same direction in health-related quality of life and capability wellbeing, with better outcomes among participants in the intervention group at both follow-up visits, the only difference that reached statistical significance was EQ-5D-5L scores (ie, quality of life) at 8 months. Importantly, our results suggest that the number of sessions received positively correlated with a reduction in depression symptomatology (PHQ-9 scores <10).

Findings from the prespecified and additional post-hoc analyses showed a substantial benefit of the psychosocial intervention compared with enhanced usual care, despite the large losses to follow-up. This benefit might have occurred due to a combination of factors, including the inevitably smaller cluster sizes (in relation to the number of clusters) that resulted from curtailment of the trial, the smaller than anticipated degree of clustering (0·011 for the primary outcome at 8 months *vs* the previous estimate of 0·030), and, crucially, the intervention having an even larger effect (18·5 percentage points) than was anticipated (15·0 percentage point difference).

To understand whether participants who did not provide outcome data might have influenced our findings, we ran a series of sensitivity analyses. Initially, we imputed missing data using multiple imputation by chained equations models, assuming that data were missing at random. To assess the sensitivity of our findings against modest departures from this assumption, we applied a weighted sensitivity analysis using the selection model approach. Estimates from both models were similar, suggesting that participants who did not provide outcome data did not substantially influence our findings.

There are other potential explanations for why the benefit of the psychosocial intervention was larger than expected. Although a 40% recovery in symptoms of depression was observed in the control group at the 8-month follow-up visit, recovery rates did not continue to improve at the 12-month follow-up visit. This finding is different to other trials, in which participants in the control group continued to improve at later follow-up visits, whereas recovery rates in the experimental group remained relatively stable.^37^ It is possible that COVID-19 mitigation measures prevented participants in the control group from receiving care that would have otherwise helped to improve their recovery from depression. Nevertheless, this situation does not undermine the findings from our study, which show the efficacy of the intervention at 8-month and 12-month follow-up assessments in a context where vulnerable older populations were forced to isolate with scarce access to health services.

Two large randomised trials also used a collaborative care psychosocial model to treat depression in older adults, one in the UK (the CASPER Plus trial)^9^ and the other in the USA (the IMPACT trial).^8^ However, comparing these three trials is difficult, given that these two trials took place in settings with divergent epidemiological, socioeconomic, and sociocultural contexts, and contrasting health systems. Additionally, the interventions were delivered by different cadres of staff, with inconsistent inclusion criteria, different forms of psychological therapies, and outcomes measured at different timepoints. Nevertheless, the interventions used in both the PROACTIVE trial and the IMPACT trial showed a marked improvement in depression outcomes at all follow-up assessments. The intervention used in the CASPER Plus trial showed an improvement in depression outcomes 4 months after randomisation only. Findings from not only the PROACTIVE trial but also the CASPER Plus and IMPACT trials showed that task sharing, by use of different staff members to deliver the psychosocial programme and collaborative care models when adapted to local contexts and health systems, can effectively treat depression among older individuals in primary care.

Besides the deviation from the protocol due to the COVID-19 pandemic, this study has several other limitations. To date, no minimal clinically important difference for the PHQ-9 has been established in Brazil or in other LMICs. In the USA, a minimal clinically important difference for changes in PHQ-9 among older people with depression suggests a decrease of at least 5 points.^28^ However, we urge caution when using minimal clinically important differences established in other countries and populations, because these differences are quite likely to differ across settings. Another limitation to the analyses in this current study is that, given that the sample size calculation was based on the overall effect on the primary outcome, the interaction effects used for the secondary, prespecified subgroup analyses should be interpreted with caution due to their low power and precision. Family members were not officially engaged in the home sessions, but we did not prevent or assess any contact between them and the CHWs. Although CHWs would normally engage with other family members whenever present, it is probable that group conversations between CHWs and participants in the intervention group were more in depth than between CHWs and participants in the control group. Furthermore, the intervention used in this trial comprised multiple components and, as such, we were unable to differentiate the relative effects of each element on participant outcomes. Finally, our intervention was not designed for individuals with cognitive impairments, or with visual or hearing problems; therefore, these individuals would probably need a different approach to treating depression. In this study, research assistants assessed signs of cognitive impairment broadly and no specific questionnaires were used. If these assistants noticed any problems, they were advised to talk to family members and study coordinators to gather more information.

Our findings have important implications for improving access to evidence-informed interventions that can potentially reduce the gap in treating depression across LMICs. The intervention was designed to be appropriate for older adults with low educational attainment. Delivering the sessions at home improved the accessibility to care of older adults with mobility problems or other disabilities. Furthermore, CHWs had a central role in delivering these home sessions, a cadre of professionals that live in the same socioeconomically deprived neighbourhoods as do the participants, do not hold a degree from higher education, and often have never received any formal training in mental health. We provided initial training and weekly supervision to at least 120 CHWs. The positive findings of this study suggest that the challenges of developing an effective intervention for older adults from low social backgrounds, as well as preparing and transferring care to a non-specialised professional, were successfully overcome. Availability and cost of these professionals might allow for similar interventions to be implemented as an affordable treatment option for older adults with depression in countries with a scarcity of health-care personnel and low budgets. A cost-effectiveness analysis of this intervention is currently being completed, and these results will be reported in a future paper, as well as findings from other analyses not prespecified in our statistical analysis plan (eg, the assessment of tobacco and alcohol use). Future research should focus on robust trials that explore how a collaborative care model to treat depression in an ageing population can be implemented at scale, especially in resource-limited settings where these interventions are needed most.

## Data sharing

The statistical analysis plan and informed consent forms are available on request from the corresponding author. The data collected for the study, including de-identified individual participant data and a data dictionary defining each field in the set, will be made available 24 months after publication. Proposals with specific aims and an analysis plan should be directed to the corresponding author.

## Declaration of interests

We declare no competing interests.
