# Supplementary material for: A task-shared, collaborative care psychosocial intervention for improving depressive symptomatology among older adults in a socioeconomically deprived area of Brazil (PROACTIVE): a pragmatic, two-arm, parallel-group, cluster-randomised controlled trial
Source: Lancet Healthy Longev. 2022 Oct;3(10):e690–702. doi: 10.1016/S2666-7568(22)00194-5 (PMC9529612; doi:10.1016/S2666-7568(22)00194-5)
Supplement: Supplementary appendix [file mmc1.pdf]

THE LANCET
Healthy Longevity

Supplementary appendix

This appendix formed part of the original submission and has been peer reviewed. We post it as supplied by the authors.

Supplement to: Scazufca M, Nakamura CA, Seward N, et al. A task-shared, collaborative care psychosocial intervention for improving depressive symptomatology among older adults in a socioeconomically deprived area of Brazil (PROACTIVE): a pragmatic, two-arm, parallel-group, cluster-randomised controlled trial. *Lancet Healthy Longev* 2022; **3:** e690–702.

Table of contents

Appendix 1: PROACTIVE programme 1

Figure S1: Description of PROACTIVE programme 1

Appendix 2: Sensitivity analyses - missing data analyses 2

Table S1: Comparison between participants with complete data and participants with missing data at the 8-month follow-up visit 3

Table S2: Comparison between participants with complete data and participants with missing data at the 12-month follow-up visit 4

Table S3: Model 1 - Adjusted odds ratios (95% CI) for different departures from the missing at random assumption, for the outcome variable of recovery from depression at 8 months assuming greater probability of the outcome being missing when depression was present 6

Table S4: Model 2 - Adjusted odds ratios (95% CI) for different departures from the missing at random assumption, for the outcome variable of recovery from depression at 12 months assuming greater probability of the outcome being missing when depression was present 6

Table S5: Comparison of adjusted estimates from multiple imputed data using MICE models, with estimates from complete case analysis for the recovery of depression at 8 months (primary outcome) and 12 months (secondary outcome) 6

Table S6: Comparison of estimates from multiple imputed data using MICE models, with estimates from complete case analysis for secondary outcomes at 8 months 6

Table S7: Comparison of estimates from multiple imputed data using MICE models, with estimates from complete case analysis for secondary outcomes at 12 months 7

Table S8: Comparison of Complier-Average Causal Effects (CACE) on the PHQ-9 mean scores at 8-month follow-up between imputed data and complete case analyses· 7

Table S9: Comparison of Complier-Average Causal Effects (CACE) on the PHQ-9 mean scores at 12-month follow-up between imputed data and complete case analyses· 7

Appendix 3: Sensitivity analyses checking for maintenance of randomisation and sub-group analyses testing for moderation 9

Table S10: Comparison of baseline demographics and baseline measures for secondary outcomes, between trial arms, for participants followed-up at the 8-month follow-up visit 9

Table S11: Comparison of baseline demographics and secondary outcome measures, between trial arms, for participants followed-up at the 12-month follow-up visit 9

Table S12: Results of likelihood ratio test for models with and without the interaction term at 8 months for the primary outcome of recovery from depression 10

Table S13: Results of likelihood ratio test for models with and without the interaction term at 12 months 10

Appendix 4: Post-hoc analysis 12

Table S14: Odds ratio for a 50% reduction in PHQ-9 scores between baseline and 8 and 12 months 12

Appendix 1: PROACTIVE programme

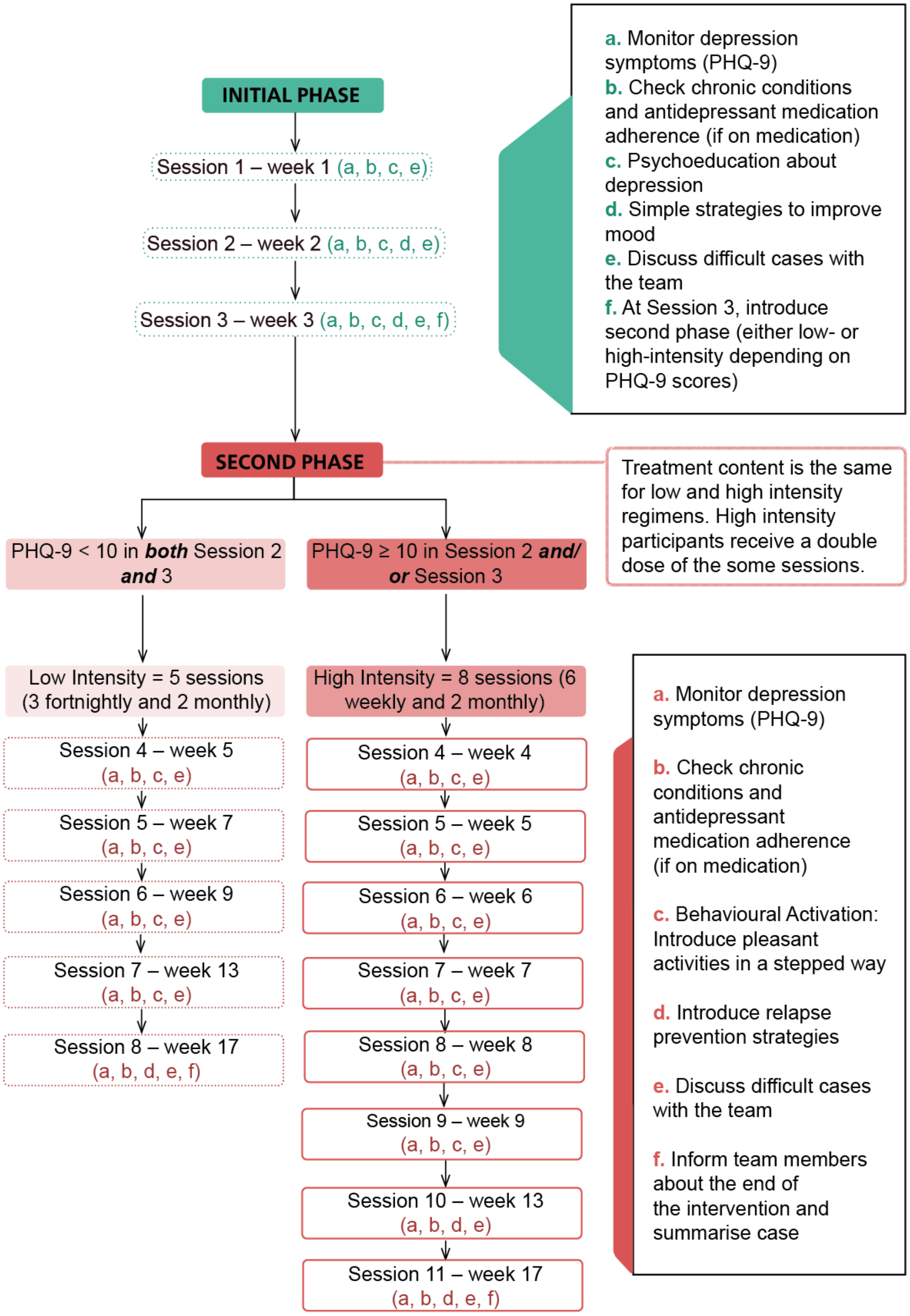

Figure S1: Description of PROACTIVE programme

Appendix 2: Sensitivity analyses - missing data analyses

Methods

Initially, patterns of missingness were investigated by comparing missing with complete data for the baseline demographic characteristics and morbidity questionnaires with the outcome of depression, by trial arm, at both follow-up visits. Given the large proportion of missingness at both the first and second follow-up visits, in order to reduce bias and loss of information, we used multiple imputation by chained equations (MICE) with 20 imputations, as implemented in the MI command in Stata under the assumption that data were missing at random (MAR).[1] Variables used in the MICE models consisted of the outcome recovered from depression, covariates described in the Statistical Analysis Plan (stratification, treatment arm, baseline PHQ-9 scores centred) and variables found to be predictors of missingness. [2,3] Predictors of missingness include the following: whether a participant had a landline or a mobile phone, whether a participant was followed-up via phone interviews after face-to-face interviews were cancelled due to the COVID-19 pandemic, and sociodemographic indicators such as gender, age, education, and income. Baseline scores for corresponding outcome models including depressive symptomatology (PHQ-9), anxiety symptomatology (GAD-7), health-related quality of life (EQ-5D-5L), and capability wellbeing (ICECAP-O) were also included.

To assess the sensitivity of our findings against modest departures from the MAR assumption, a weighted sensitivity analysis using the Selection Model Approach was applied.[4-6] Briefly, once data had been imputed under MAR, parameter estimates from each imputed dataset were reweighted to allow for the data to be missing not at random (MNAR). To test the stability of our model, we considered different degrees of departure from the MAR assumption by considering plausible values of δ ranging from 0·10 to 0·40. This range corresponds to odds ratios for the data being observed when a participant recovered from depression compared to when it did not, ranging from 1·11 to 1·50 (i.e. exponential of 0·10 and 0·40).

Results

General

Tables 1 and 2 demonstrate findings from the analysis comparing baseline demographics and baseline measures for the secondary outcomes between participants with complete data, and those with missing data, at 8- and 12-months follow-up visits respectively.

Table S1: Comparison between participants with complete data and participants with missing data at the 8-month follow-up visit

	PROACTIVE				Enhanced Usual Care			
	PHQ>10 (n=95)	PHQ<10 (n=158)	Total (n=253)	Missing PHQ-9 (n=107)	PHQ>10 (n=159)	PHQ<10 (n=125)	Total (n=284)	Missing PHQ-9 (n=71)
**Landline**								
No	43 (45·3)	60 (38·0)	103 (40·7)	57 (53·8)	65 (41·1)	40 (32·3)	105 (37·2)	40 (56·3)
Yes	52 (54·7)	98 (62·0)	150 (59·3)	49 (46·2)	93 (58·9)	84 (67·7)	177 (62·8)	31 (43·7)
**Mobile phone**								
No	36 (37·9)	48 (30·4)	84 (33·2)	49 (46·2)	59 (37·3)	45 (36·3)	104 (36·9)	34 (47·9)
Yes	59 (62·1)	110 (69·6)	169 (66·8)	57 (53·8)	99 (62·7)	79 (63·7)	178 (63·1)	37 (52·1)
**Gender**								
Male	13 (13·7)	45 (28·5)	58 (22·9)	34 (31·8)	36 (22·6)	33 (26·4)	69 (24·3)	24 (33·8)
Female	82 (86·3)	113 (71·5)	195 (77·1)	73 (68·2)	123 (77·4)	92 (73·6)	215 (75·7)	47 (66·2)
**Age group**								
60-69	65 (68·4)	99 (62·7)	164 (64·8)	59 (55·1)	97 (61·0)	77 (61·6)	174 (61·3)	43 (60·6)
70-79	23 (24·2)	51 (32·3)	74 (29·3)	36 (33·6)	47 (29·6)	40 (32·0)	87 (30·6)	17 (23·9)
80+	7 (7·4)	8 (5·1)	15 (5·9)	12 (11·2)	15 (9·4)	8 (6·4)	23 (8·1)	11 (15·5)
**Education**								
None	16 (16·8)	33 (20·9)	49 (19·4)	23 (21·7)	30 (19·0)	22 (17·6)	52 (18·4)	13 (18·3)
1-4 years	50 (52·6)	72 (45·6)	122 (48·2)	51 (48·1)	81 (51·3)	61 (48·8)	142 (50·2)	38 (53·5)
5-8 years	18 (19·0)	32 (20·3)	50 (19·8)	20 (18·9)	31 (19·6)	25 (20·0)	56 (19·8)	15 (21·1)
>8 years	11 (11·6)	21 (13·3)	32 (12·6)	12 (11·3)	16 (10·1)	17 (13·6)	33 (11·7)	5 (7·0)
**Personal income**								
Up to 1 MW	63 (70·0)	116 (76·8)	179 (74·3)	84 (80·0)	116 (77·3)	88 (74·6)	204 (76·1)	51 (73·9)
>1-2 MW	21 (23·3)	22 (14·6)	43 (17·8)	18 (17·1)	20 (13·3)	21 (17·8)	41 (15·3)	14 (20·3)
>2+ MW	6 (6·7)	13 (8·6)	19 (7·9)	3 (2·9)	14 (9·3)	9 (7·6)	23 (8·6)	4 (5·8)
**Hypertension**								
No hypertension	23 (24·2)	40 (25·3)	63 (24·9)	23 (21·5)	35 (22·0)	29 (23·2)	64 (22·5)	23 (32·4)
Hypertension	72 (75·8)	118 (74·7)	190 (75·1)	84 (78·5)	124 (78·0)	96 (76·8)	220 (77·5)	48 (67·6)
**Diabetes**								
No diabetes	56 (59·0)	90 (57·0)	146 (57·7)	66 (61·7)	89 (56·0)	72 (57·6)	161 (56·7)	48 (67·6)
Diabetes	39 (41·0)	68 (43·0)	107 (42·3)	41 (38·3)	70 (44·0)	53 (42·4)	123 (43·3)	23 (32·4)
**PHQ-9 score at baseline, M (SD)**	17·09 (4·89)	15·28 (4·15)	15·96 (4·52)	16·22 (4·72)	17·34 (4·89)	14·77 (4·22)	16·21 (4·78)	16·85 (4·34)
**GAD-7 score at baseline, M (SD)**	12·17 (5·48)	9·31 (6·06)	10·39 (6·00)	9·63 (6·20)	10·90 (6·22)	7·46 (5·17)	9·40 (6·03)	9·54 (6·70)
**EQ-5D-5L score at baseline, M (SD)**	0·726 (0·196)	0·786 (0·197)	0·764 (0·198)	0·747 (0·224)	0·723 (0·193)	0·803 (0·164)	0·761 (0·184)	0·719 (0·240)
**ICECAP-O score at baseline, M (SD)**	0·602 (0·225)	0·666 (0·167)	0·642 (0·193)	0·593 (0·203)	0·615 (0·211)	0·672 (0·163)	0·640 (0·193)	0·636 (0·218)

Abbreviations: EQ-5D-5L: 5-level EuroQol health-related quality of life questionnaire; GAD-7: 7-item General Anxiety Disorder questionnaire; ICECAP-O: ICEpop CAPability measure for older people; M: mean; MW: minimum wage; PHQ-9: 9-item Patient Health Questionnaire for depression; SD: standard deviation
PHQ-9 scores range from 0 to 27, with higher scores representing more severe symptoms of depression
GAD-7 scores ranges from 0 to 21 with higher scores representing more severe symptoms of anxiety
EQ-5D-5L scores range from -0·264 to 1, with higher scores representing higher quality of life
ICECAP-O scores range from 0 to 1, with higher scores representing greater levels of capability wellbeing

Table S2: Comparison between participants with complete data and participants with missing data at the 12-month follow-up visit

	**PROACTIVE**				**Enhanced Usual Care**			
	PHQ>10 (n=78)	PHQ<10 (n=115)	Total (n=193)	Missing PHQ-9 (n=167)	PHQ>10 (n=111)	PHQ<10 (n=77)	Total (n=188)	Missing PHQ-9 (n=167)
**Recovery at 8 months**								
No	43 (59·7)	22 (20·8)	65 (36·5)	30 (40·0)	73 (68·9)	30 (41·1)	103 (57·5)	56 (53·3)
Yes	29 (40·3)	84 (79·2)	113 (63·5)	45 (60·0)	33 (31·1)	43 (58·9)	76 (42·5)	49 (46·7)
**Landline**								
No	34 (43·6)	45 (39·1)	79 (40·9)	81 (48·8)	42 (37·8)	28 (36·4)	70 (37·2)	75 (45·5)
Yes	44 (56·4)	70 (60·9)	114 (59·1)	85 (51·2)	69 (62·2)	49 (63·6)	118 (62·8)	90 (54·5)
**Mobile phone**								
No	24 (30·8)	33 (28·7)	57 (29·5)	76 (45·8)	37 (33·3)	25 (32·5)	62 (33·0)	76 (46·1)
Yes	54 (69·2)	82 (71·3)	136 (70·5)	90 (54·2)	74 (66·7)	52 (67·5)	126 (67·0)	89 (53·9)
**Gender**								
Male	14 (17·9)	31 (27·0)	45 (23·3)	47 (28·1)	25 (22·5)	21 (27·3)	46 (24·5)	47 (28·1)
Female	64 (82·1)	84 (73·0)	148 (76·7)	120 (71·9)	86 (77·5)	56 (72·7)	142 (75·5)	120 (71·9)
**Age group**								
60-69	50 (64·1)	83 (72·2)	90 (53·9)	133 (68·9)	69 (62·2)	48 (62·3)	117 (62·2)	100 (59·9)
70-79	21 (26·9)	26 (22·6)	63 (37·7)	47 (24·4)	35 (31·5)	23 (29·9)	58 (30·9)	46 (27·5)
80+	7 (9·0)	6 (5·2)	14 (8·4)	13 (6·7)	7 (6·3)	6 (7·8)	13 (6·9)	21 (12·6)
**Education**								
None	8 (10·3)	19 (16·5)	27 (14·0)	45 (27·1)	16 (14·4)	15 (19·5)	31 (16·5)	34 (20·5)
1-4 years	39 (50·0)	56 (48·7)	95 (49·2)	78 (47·0)	60 (54·1)	39 (50·6)	99 (52·7)	81 (48·8)
5-8 years	19 (24·4)	26 (22·6)	45 (23·3)	25 (15·1)	23 (20·7)	14 (18·2)	37 (19·7)	34 (20·5)
>8 years	12 (15·4)	14 (12·2)	26 (13·5)	18 (10·8)	12 (10·8)	9 (11·7)	21 (11·2)	17 (10·2)
**Personal income**								
Up to 1 MW	55 (72·4)	83 (76·1)	138 (74·6)	125 (77·6)	79 (75·2)	58 (80·6)	137 (77·4)	118 (73·8)
>1-2 MW	17 (22·4)	19 (17·4)	36 (19·5)	25 (15·5)	21 (20·0)	7 (9·7)	28 (15·8)	27 (16·9)
>2+ MW	4 (5·3)	7 (6·4)	11 (5·9)	11 (6·8)	5 (4·8)	7 (9·7)	12 (6·8)	15 (9·4)
**Hypertension**								
No hypertension	20 (25·6)	30 (26·1)	50 (25·9)	36 (21·6)	23 (20·7)	23 (29·9)	46 (24·5)	41 (24·6)
Hypertension	58 (74·4)	85 (73·9)	143 (74·1)	131 (78·4)	88 (79·3)	54 (70·1)	142 (75·5)	126 (75·4)
**Diabetes**								
No diabetes	44 (56·4)	61 (53·0)	105 (54·4)	107 (64·1)	60 (54·1)	48 (62·3)	108 (57·4)	101 (60·5)
Diabetes	34 (43·6)	54 (47·0)	88 (45·6)	60 (35·9)	51 (45·9)	29 (37·7)	80 (42·6)	66 (39·5)
**PHQ-9 score at baseline, M (SD)**	17·82 (4·74)	15·12 (4·10)	16·21 (4·56)	15·84 (4·60)	17·07 (4·61)	14·34 (4·05)	15·95 (4·58)	16·77 (4·80)
**GAD-7 score at baseline, M (SD)**	12·36 (5·52)	9·09 (6·24)	10·42 (6·16)	9·87 (5·96)	11·26 (5·91)	6·65 (5·41)	9·37 (6·13)	9·49 (6·12)

**EQ-5D-5L score at baseline, M (SD)**	0·716 (0·199)	0·804 (0·188)	0·768 (0·197)	0·747 (0·216)	0·726 (0·204)	0·808 (0·153)	0·760 (0·189)	(0·745 (0·205)
**ICECAP-O score at baseline, M (SD)**	0·612 (0·203)	0·667 (0·188)	0·653 (195)	0·598 (196)	0·615 (190)	0·686 (189)	0·644 (0·192)	0·633 (0·205)

Abbreviations: EQ-5D-5L: 5-level EuroQol health-related quality of life questionnaire; GAD-7: 7-item General Anxiety Disorder questionnaire; ICECAP-O: ICEpop CAPability measure for older people; M: mean; MW: minimum wage; PHQ-9: 9-item Patient Health Questionnaire for depression; SD: standard deviation
PHQ-9 scores range from 0 to 27, with higher scores representing more severe symptoms of depression
GAD-7 scores ranges from 0 to 21 with higher scores representing more severe symptoms of anxiety
EQ-5D-5L scores range from -0·264 to 1, with higher scores representing higher quality of life
ICECAP-O scores range from 0 to 1, with higher scores representing greater levels of capability wellbeing

Sensitivity analysis checking the missing at random (MAR) assumption for the primary outcome

Results from the sensitivity analysis testing the MAR assumption for the primary outcome of recovery from depression at 8 months suggest that estimates moved towards the null. However at 12 months, the opposite occurred, suggesting a different mechanism for missing data (participants who recovered from depression, more likely to be missing at follow-up, compared to participants who did not recover from depression). Regardless of the mechanism, results remained highly significant.

Table S3: Model 1 - Adjusted odds ratios (95% CI) for different departures from the missing at random assumption, for the outcome variable of recovery from depression at 8 months assuming greater probability of the outcome being missing when depression was present

Delta	Adjusted Odds Ratio (95% CI)
0·1	2·11 (1·50, 2·97)
0·2	2·11 (1·50, 2·97)
0·3	2·08 (1·49, 2·91)
0·4	2·08 (2·90, 2·90)

Table S4: Model 2 - Adjusted odds ratios (95% CI) for different departures from the missing at random assumption, for the outcome variable of recovery from depression at 12 months assuming greater probability of the outcome being missing when depression was present

Delta	Adjusted Odds Ratio (95% CI)
0·1	2·44 (1·68, 3·54)
0·2	2·47 (1·52, 4·00)
0·3	2·51 (1·76, 3·56)
0·4	2·54 (1·82, 3·57)

Results comparing estimates from MICE models with complete case analyses for both primary and secondary outcomes

Table S5: Comparison of adjusted estimates from multiple imputed data using MICE models, with estimates from complete case analysis for the recovery of depression at 8 months (primary outcome) and 12 months (secondary outcome)

	**Complete case analysis [a,b,c]**		**Imputed data from MICE analyses (N=715) [a,b,c]**	
**Model**	**Odds ratio (95% CI)**	***p value***	**Odds ratio (95% CI)**	***p value***
Recovery from depression at 8 months [a, b] (n=537)	2·17 (1·46, 3·32)	<0·0001	2·16 (1·47, 3·18)	<0·0001
Recovery from depression at 12 months [a,b] (n=381)	2·38 (1·54, 3·37)	<0·0001	2·33 (1·45, 3·71)	<0·0001

[a] The primary and secondary outcome of recovery from depression was defined as PHQ-9 scores less than 10

[b] Odds ratios and 95% CIs were calculated using random effects logistic regression models adjusted for relevant baseline assessment of PHQ-9 scores and stratified variable of education

[c] All estimates had missing data imputed by trial arm using MICE models that included baseline PHQ-9 scores, stratification variables and predictors of missingness

Table S6: Comparison of estimates from multiple imputed data using MICE models, with estimates from complete case analysis for secondary outcomes at 8 months

	**Complete case analysis [a,b]**		**Imputed data from MICE analyses (N=715) [a,b]**	
**Model**	**Difference in means (95% CI)**	***p value***	**Difference in means (95% CI)**	***p value***
PHQ-9 scores [a]	-2·58 (-3·87, -1·30)	<0·0001	-2·52 (-3·69, -1·36)	<0·0001
GAD-7 scores [a]	-1·25 (-2·48, -0·14)	0·047	-1·21 (-2·29, -0·12)	0·030
EQ-5D-5L scores [a]	0·028 (0·000, 0·057)	0·050	0·030 (0·003, 0·056)	0·029
ICECAP-O scores [a]	0·021 (-0·014, 0·056)	0·24	0·014 (-0·015, 0·043)	0·34

[a] Differences in means were estimated using linear regression models with random effects for the cluster randomised trial, adjusted for relevant baseline assessment of corresponding outcome and the stratified variable of education

[b] All estimates had missing data imputed seperately, by trial arm, using MICE models that included predictors of missingness such as stratification, baseline PHQ-9 scores, and corresponding baseline value for outcome in question.

PHQ-9 scores range from 0 to 27, with higher scores representing more severe symptoms of depression

GAD-7 scores ranges from 0 to 21 with higher scores representing more severe symptoms of anxiety

EQ-5D-5L scores range from -0·264 to 1, with higher scores representing higher quality of life

ICECAP-O scores range from 0 to 1, with higher scores representing greater levels of capability wellbeing

Table S7: Comparison of estimates from multiple imputed data using MICE models, with estimates from complete case analysis for secondary outcomes at 12 months

	**Complete case analysis [a,b]**		**Imputed data from MICE analyses (N=715) [a,b]**	
**Model**	**Difference in means (95% CI)**	***p value***	**Difference in means (95% CI)**	***p value***
PHQ-9 scores [a]	-2·37 (-3·72, -1·02)	0·0010	-2·32 (-4·08, -0·55)	0·011
GAD-7 scores [a]	-1·79 (-3·09, -0·485)	0·0070	-2·09 (-3.30, -0.89)	0·0010
EQ-5D-5L scores [a]	0·003 (-0·028, 0·034)	0·86	0·003 (-0·028, 0·035)	0·83
ICECAP-O scores [a]	0·045 (0·004, 0·086)	0·030	0·039 (-0·011, 0·090)	0·12

[a] Differences in means were estimated using linear regression models with random effects for the cluster randomised trial, adjusted for relevant baseline assessment of corresponding outcome and the stratified variable of education

[b] All estimates had missing data imputed seperately, by trial arm, using MICE models that included predictors of missingness, stratification, baseline PHQ-9 scores, corresponding baseline value for outcome in question

PHQ-9 scores range from 0 to 27, with higher scores representing more severe symptoms of depression

GAD-7 scores ranges from 0 to 21 with higher scores representing more severe symptoms of anxiety

EQ-5D-5L scores range from -0·264 to 1, with higher scores representing higher quality of life

ICECAP-O scores range from 0 to 1, with higher scores representing greater levels of capability and general wellbeing

Table S8: Comparison of Complier-Average Causal Effects (CACE) on the PHQ-9 mean scores at 8-month follow-up between imputed data and complete case analyses·

	**Complete case (n=537) [a,b]**		**Imputed data from MICE analyses (N=715) [a,b,c]**	
**Threshold**	**Adjusted difference in PHQ-9 means (95% CI)**	***p* value**	**Adjusted difference in PHQ-9 means (95% CI)**	***p* value**
3 sessions	-4·46 (-6·41, -2·51)	<0·0001	-5·09 (-7·40, -2·77)	<0·0001
4 sessions	-5·25 (-7·59, -2·89)	<0·0001	-6·05 (-8·85, -3·26)	<0·0001
5 sessions	-6·19 (-8·99, -3·38)	0·0010	-7·23 (-10·61, -3·85)	<0·0001

[a] All models are adjusted for baseline PHQ-9 and stratification·

[b] Standard errors and confidence intervals adjusted for clustering

[c] All estimates had missing data imputed seperately, by trial arm, using MICE models that included predictors of missingness, stratification, baseline PHQ-9 scores

Table S9: Comparison of Complier-Average Causal Effects (CACE) on the PHQ-9 mean scores at 12-month follow-up between imputed data and complete case analyses·

	**Complete case (n=341) [a,b]**		**Imputed data from MICE analyses (N=715) [a,b,c]**	
**Threshold**	**Adjusted difference in PHQ-9 means (95% CI)**	***p* value**	**Adjusted difference in PHQ-9 means (95% CI)**	***p* value**
3 sessions	-3·82 (-5·99, -1·65)	0·0010	-4·93 (-7·89, -1·97)	0·0010
4 sessions	-4·58 (-7·23, -1·93)	<0·0001	-5·86 (-9·41, -2·31)	0·0020
5 sessions	-5·67 (-8·97, -2·37)	0·0010	-7·00 (-11·27, -2·74)	0·0020

[a] All models are adjusted for baseline PHQ-9 and stratification·

[b] Standard errors and confidence intervals adjusted for clustering

[c] All estimates had missing data imputed seperately, by trial arm, using MICE models that included predictors of missingness stratification, baseline PHQ-9 scores

Table S10 Comparison of Complier-Average Causal Effects (CACE) on PHQ-9 mean scores at 8-month and 12 months follow-up between imputed data and complete case analyses for participants who completed at least four sessions, regardless of whether this was by phone or face to face

	**Complete case [a,b]**		**Imputed data from MICE analyses (N=715) [a,b,c]**	
**Visit**	**Adjusted difference in PHQ-9 means (95% CI)**	***p* value**	**Adjusted difference in PHQ-9 means (95% CI)**	***p* value**
8 months (complete cases: n=537)	-4·62 (-6·65, -2·59)	0·0010	-5·42 (-7·89, -2·95)	<0·0001
12 months (complete cases: n=341	-4·05 (-6·37, -1·72)	0·0010	-5·25 (-8·42, -2·08)	0·0010

[a] All models are adjusted for baseline PHQ-9 and stratification·

[b] Standard errors and confidence intervals adjusted for clustering

[c] All estimates had missing data imputed seperately, by trial arm, using MICE models that included predictors of missingness stratification, baseline PHQ-9 scores

References

1. Lee KJ, Carlin JB. Multiple imputation for missing data: fully conditional specification versus multivariate normal imputation. Am J Epidemiol. 2010;171(5):624-32.
2. Sterne JA, White IR, Carlin JB, et al. Multiple imputation for missing data in epidemiological and clinical research: potential and pitfalls. BMJ. 2009;338:b2393.
3. Rubin DB. Multiple Imputation for Nonresponse in Surveys. Wiley Series in Probability and Statistics. New York: John Wiley & Sons, Inc., 1987.
4. Carpenter J, Kenward M, White I. Sensitivity analysis after multiple imputation under missing at random: a weighting approach. Statistical methods in medical research. 2007;16(3):259-75.
5. Carpenter J, Pocock S, Lamm C. Coping with missing data in clinical trials: a model-based approach applied to asthma trials. Stat Med. 2002;21(8):1043-66.
6. Heraud-Bousquet V, Larsen C, Carpenter J, Desenclos JC, Le Strat Y. Practical considerations for sensitivity analysis after multiple imputation applied to epidemiological studies with incomplete data. BMC medical research methodology. 2012;12:73.

Appendix 3: Sensitivity analyses checking for maintenance of randomisation and sub-group analyses testing for moderation

Sensitivity analysis to determine if randomisation was maintained at the 8- and 12- month follow-up visits
The comparison at the 8-month (Table 1) and 12-month follow-up (Table 2) visits suggests a slight imbalance whereby participants in the intervention arm were slightly younger and received more pharmacological treatment for depression compared with participants in the control arm. Additionally, at the 12-month follow-up visit participants in the intervention arm also had slightly higher levels of education, higher levels of participants that reported having diabetes, and higher levels anxiety symptomatology indicated by the GAD-7 score. However, none of these observed differences were deemed to be sufficiently large to warrant adjustment in the relevant secondary analyses.

Table S10: Comparison of baseline demographics and baseline measures for secondary outcomes, between trial arms, for participants followed-up at the 8-month follow-up visit

	**PROACTIVE (n=253)**	**Enhanced Usual Care (n=284)**
**Female, No. (%)**	195 (77·1)	215 (75·7)
**Age group, No. (%)**		
60-69	164 (64·8)	174 (61·3)
70-79	74 (29·2)	87 (30·6)
80+	15 (5·9)	23 (8·1)
**Education, No. (%)**		
None	49 (19·4)	52 (18·4)
1-4 years	122 (48·2)	142 (50·2)
5-8 years	50 (19·8)	56 (19·8)
>8 years	32 (12·6)	33 (11·7)
Missing	*0 [0·0]*	*1 [0·4]*
**Personal income, No. (%)**		
Up to 1 MW	179 (74·3)	204 (76·1)
>1-2 MW	43 (17·8)	41 (15·3)
>2 MW	19 (7·9)	23 (8·6)
Missing	*12 [4·7]*	*16 [5·6]*
**Hypertension (self-reported), No (%)**	190 (75·1)	220 (77·5)
**Diabetes (self-reported), No (%)**	107 (42·3)	123 (43·3)
**Receiving pharmacological treatment for depression (self-reported), No (%)**		
No	206 (82·4)	235 (83·3)
Yes	44 (17·6)	47 (16·7)
Missing	*3 [1·2]*	*2 [0·7]*
**PHQ-9 score, M (SD)**	15·96 (4·52)	16·21 (4·78)
**PHQ-9 score, Median (IQR)**	15 (12, 19)	15 (12, 20)
**GAD-7 score, M (SD)** ***[No. of missing cases]***	10·39 (6·00) *[1]*	9·40 (6·03) *[2]*
**GAD-7 score, Median (IQR)**	11 (5, 15)	9 (4, 14)
**EQ-5D-5L score, M (SD)** ***[No. of missing cases]***	0·764 (0·198) *[1]*	0·761 (0·184) *[0]*
**EQ-5D-5L score, Median (IQR)**	0·827 (0·663, 0·909)	0·812 (0·666, 0·904)
**ICECAP-O score, M (SD)** ***[No. of missing cases]***	0·642 (0·193) *[3]*	0·640 (0·193) *[0]*
**ICECAP-O score, Median (IQR)**	0·663 (0·522, 0·794)	0·669 (0·538, 0·778)

Abbreviations: EQ-5D-5L: 5-level EuroQol health-related quality of life questionnaire; GAD-7: 7-item General Anxiety Disorder questionnaire; ICECAP-O: ICEpop CAPability measure for older people; M: mean; MW: minimum wage; No.: number of cases; PHQ-9: 9-item Patient Health Questionnaire for depression; SD: standard deviation. Italised numbers represent missing cases.
PHQ-9 scores range from 0 to 27, with higher scores representing more severe symptoms of depression
GAD-7 scores ranges from 0 to 21 with higher scores representing more severe symptoms of anxiety
EQ-5D-5L scores range from -0·264 to 1, with higher scores representing higher quality of life
ICECAP-O scores range from 0 to 1, with higher scores representing greater levels of capability wellbeing

Table S11: Comparison of baseline demographics and secondary outcome measures, between trial arms, for participants followed-up at the 12-month follow-up visit

**Baseline variables**	**PROACTIVE (n=193)**	**Enhanced Usual Care (n=188)**
**Female, No. (%)**	148 (76·7)	142 (75·5)
**Age group, No. (%)**		
60-69	133 (68·9)	117 (62·2)
70-79	47 (24·4)	58 (30·9)

80+	13 (6·7)	13 (6·9)
**Education, No. (%)**		
None	27 (14·0)	31 (16·5)
1-4 years	95 (49·2)	99 (52·7)
5-8 years	45 (23·3)	37 (19·7)
>8 years	26 (13·5)	21 (11·2)
Missing	*0 [0·0]*	*1 [0·4]*
**Personal income, No. (%)**		
Up to 1 MW	138 (71·5)	137 (72·9)
>1-2 MW	36 (18·7)	28 (14·9)
>2 MW	11 (5·7)	12 (6·4)
Missing	*8 (4·2)*	*11 (5·9)*
**Hypertension (self-reported), No (%)**	143 (74·1)	142 (75·5)
**Diabetes (self-reported), No (%)**	88 (45·6)	80 (42·6)
**Receiving pharmacological treatment for depression (self-reported), No (%)**		
No	155 (80·3)	158 (84·0)
Yes	36 (18·7)	29 (15·4)
Missing	*2 (1·0)*	*1 (0·5)*
**PHQ-9 score, M (SD)**	15·84 (4·60)	16·77 (4·80)
**PHQ-9 score, Median (IQR)**	15 (12, 19)	15 (12, 20)
**GAD-7 score, M (SD)** ***[No. of missing cases]***	10·41 (6·15) *[1]*	9·37 (6·13) *[0]*
**GAD-7 score, Median (IQR)**	11 (5, 16)	9 (4, 14)
**EQ-5D-5L score, M (SD)** ***[No. of missing cases]***	0·768 (0·197) *[1]*	0·759 (0·189) *[0]*
**EQ-5D-5L score, Median (IQR)**	0·827 (0·681, 0·909)	0·818 (0·679, 0·896)
**ICECAP-O score, M (SD)** ***[No. of missing cases]***	0·653 (0·195) *[2]*	0·644 (0·192) *[0]*
**ICECAP-O score, Median (IQR)**	0·680 (0·537, 0·797)	0·672 (0·545, 0·774)

Abbreviations: EQ-5D-5L: 5-level EuroQol health-related quality of life questionnaire; GAD-7: 7-item General Anxiety Disorder questionnaire; ICECAP-O: ICEpop CAPability measure for older people; M: mean; MW: minimum wage; No.: number of cases; PHQ-9: 9-item Patient Health Questionnaire for depression; SD: standard deviation. Italised numbers represent missing cases.

PHQ-9 scores range from 0 to 27, with higher scores representing more severe symptoms of depression

GAD-7 scores ranges from 0 to 21 with higher scores representing more severe symptoms of anxiety

EQ-5D-5L scores range from -0·264 to 1, with higher scores representing higher quality of life

ICECAP-O scores range from 0 to 1, with higher scores representing greater levels of capability wellbeing

Moderation by pre-specified variables for either primary or secondary outcomes

None of the pre-specified subgroup analyses performed to investigate the potential differential intervention effects on the primary outcome of recovery was found to be relevant based on the significance of the corresponding likelihood ratio tests. Table S12 shows the results of the likelihood ratio statistic for models with and without the interaction term at 8 months, and Table S13 shows the results at 12 months. Multiplicative interactions were assessed on the log odds scale.

Table S12: Results of likelihood ratio test for models with and without the interaction term at 8 months for the primary outcome of recovery from depression

Interaction term with treatment allocation	*p* value of LRT
Gender	0·090
Age group	0·39
Education	0·90
Baseline PHQ-9 scores	0·44
Diabetes	0·95
Hypertension	0·80
Comorbid hypertension and diabetes	0·70

Abbreviations: LRT: likelihood ratio test; PHQ-9: 9-item Patient Health Questionnaire for depression.

Table S13: Results of likelihood ratio test for models with and without the interaction term at 12 months

Interaction term with treatment allocation	*p* value of LRT
Gender	0·68
Age group	0·67
Education	0·61
Baseline PHQ-9 scores	0·89
Diabetes diagnosis	0·65

Hypertension diagnosis	0·24
Comorbid hypertension and diabetes	0·11

Abbreviations: LRT: likelihood ratio test; PHQ-9: 9-item Patient Health Questionnaire for depression.

Sensitivity analyses checking for potential effect between mean number of elapsed days between baseline and the first follow-up visit

An additional analysis was performed to explore the potential effect of differences in the mean number of days elapsed from baseline to the first follow-up between the intervention arm (median number of days (MDN)=228, intra quartile range (IQR) 218-237) and control arm (MDN=225, IQR 216-245). Estimates from models using complete cases only, that adjusted for mean number of days, were very similar (Odds ratio for difference in recovery from depression at eight months between intervention and control arm: 2·25; 95% CI: 1·49, 3·39), to the model that did not adjust for mean number of days (2·17, 1·46, 3·23).

Adjusted models accounting for ‘type of follow-up visit’

Results from the adjusted model that included ‘type of follow-up visit’ [difference between mean EQ-5D-5L scores across trial arms (0·027; 95% CI: 0·001, 0·053)] were similar to estimates from a model that did not include ‘type of follow-up visit’ (0·029; 95% CI: 0, 0·057). However, results from the ANOVA model comparing EQ-5D-5L scores by type of follow-up visit, found that scores were significantly higher ($p<0·014$) when interviews were conducted by phone (mean EQ-5D-5L score: 0·827, SD (0·0166)) compared to interviews that were conducted face to face (0·779 (0·209).

Appendix 4: Post-hoc analysis

Additional post-hoc analysis were conducted to estimate depression outcomes not pre-specified in our Statistical Analysis Plan. The following models used complete case analysis.

Modified Poisson regression models

We ran an analysis using the modified Poisson regression models suggested by Zou and Donner[1] with complete data only, and adjusting for the same factors as our models used to assess for the primary outcome. Our results remained highly significant using this approach (Incidence rate ratio 1·41, 95% CI 1·20, 1·64). However, the estimates were moved towards the null, compared to the findings using mixed effects logistic regression models using complete data only (Appendix 2, Table S5: Odds Ratio 2·17: 95% CI: 1·46, 3·32).

Models using the outcome of a reduction in PHQ-9 scores between baseline and the 8th and 12 months follow-up visit

We also compared estimates from using our pre-determined outcome of recovery from depression at 8th and 12 months (PHQ-9 <10) with the outcome of a 50% reduction in PHQ-9 scores between baseline and 8 and 12 months.

Table S14: Odds ratio for a 50% reduction in PHQ-9 scores between baseline and 8 and 12 months

Time period	Odds ratio (95% CI)
8th month follow-up	1·75 (1·25, 2·47)
12th month follow-up	1·96 (1·28, 3·00)

*models were adjusted for similar variables as the models assessing the primary outcome in the main analysis

References

1. Zou GY, Donner A. Extension of the modified Poisson regression model to prospective studies with correlated binary data. Stat Methods Med Res. Dec 2013;22(6):661-70. doi:10.1177/0962280211427759
